# Pain‐Related Outcome Domains and Measures in Randomized Controlled Trials of Treatment for Acute Abdominal Pain: A Systematic Review

**DOI:** 10.1002/ejp.70445

**Published:** 2026-11-04

**Authors:** Louise Kuhlmann, Cecilie Siggaard Knoph, Søren Schou Olesen, Nejo Joseph, John Windsor, Asbjørn M. Drewes, Sanjay Pandanaboyana, Esther Pogatzki‐Zahn

**Affiliations:** ^1^ Center for Pancreatic Diseases & Mech‐Sense, Department of Gastroenterology and Hepatology Aalborg University Hospital Aalborg Denmark; ^2^ Department of Clinical Medicine Aalborg University Aalborg Denmark; ^3^ Department of Surgery University of Auckland Auckland New Zealand; ^4^ Department of HPB and Transplant Surgery Freeman Hospital Newcastle Upon Tyne UK; ^5^ Population Health Sciences Institute Newcastle University Newcastle UK; ^6^ Department of Anesthesiology, Intensive Care and Pain Medicine University Hospital Muenster Muenster Germany

## Abstract

**Background and Objectives:**

Acute abdominal pain frequently presents to emergency departments, yet outcome domains in randomized controlled trials (RCTs) remain poorly characterized. This review identified pain‐related domains and outcome measures used in RCTs on acute abdominal pain.

**Databases and Data Treatment:**

This PRISMA‐guided systematic review was registered in PROSPERO (CRD42025630171). PubMed, Embase, Scopus, and Web of Science were searched for RCTs (January 2000–February 2025). Eligible studies included adult and paediatric patients with acute abdominal pain reporting pain assessment. Outcome domains extracted included pain intensity, rescue analgesia, adverse effects, pain interference, quality of life (QoL), and psychological function. Bias was assessed using Cochrane RoB2.

**Results:**

Of 826 records, 43 RCTs were included; eight included children. In adult trials, pain intensity was assessed using the visual analogue scale (*n* = 19) or the numeric rating scale (*n* = 15). Rescue analgesia (44%) and adverse effects (84%) were frequently reported. No study assessed pain interference or psychological function, and QoL was listed as an outcome in one trial. All were conducted in emergency settings, with short‐term follow‐up within the first hours.

**Conclusions:**

Pain assessment in acute abdominal pain RCTs is predominantly limited to intensity measures and short‐term outcomes. While appropriate for immediate analgesic effects, this approach may be insufficient when broader treatment goals or longer follow‐up are relevant. Context‐specific outcome selection is needed, aligning domains with clinical purpose and time frame.

**Significance:**

RCTs on acute abdominal pain assess pain incompletely, focusing predominantly on intensity rather than broader domains such as pain interference and psychological function. None adhered to the INTEGRATE framework for pain assessment. This review provides the first systematic mapping of outcome domains in this area, offering a foundation for context‐specific outcome selection aligned with what matters to patients.

## 1 Introduction

Abdominal pain is the most common reason for contact in emergency departments, accounting for approximately 9% of all visits in the United States (Wu et al. 2025). It ranges from mild diffuse discomfort to severe localized pain, with sensory, affective, and autonomic components (Cervero 2010; Drewes et al. 2020; Xiao and Zhang 2018). Accurate assessment is essential for both diagnosis and pain management, yet abdominal pain can be diagnostically ambiguous: it may originate from multiple visceral or somatic sources, its intensity does not reliably correlate with disease severity (Cervero 1991; Shaballout et al. 2021), and reporting is shaped by psychological factors (Vassend et al. 2013). Clinically, the need for timely analgesia must be balanced against the risk of obscuring diagnostic signs (Villain et al. 2013).

Management of acute abdominal pain remains suboptimal. Adequate analgesia and improved mobilization are often not achieved, and clinical trial evidence does not consistently translate into practice, partly due to mixed findings from randomized controlled trials (RCTs) (Chambers 2018). A key contributing factor is the absence of a comprehensive, standardized approach to pain assessment.

Using a Core Outcome Set (COS) for acute abdominal pain would address this gap. A COS defines the minimum outcomes that should be assessed across all clinical trials and clinical practice for a specific condition, agreed upon by relevant stakeholders including patients (Williamson et al. 2017). Per the Core Outcome Measures in Effectiveness Trials (COMET) initiative, outcome selection should follow a construct‐first principle: domains (e.g., pain intensity, pain interference, physical function) must be defined before selecting measurement instruments, which should be chosen based on construct relevance and measurement properties rather than convenience (Mokkink et al. 2016). A recently developed COS for acute pain in general recommends extending assessment beyond pain intensity to include, at minimum, pain interference, physical function, and quality of life (Bova et al. 2023, 2025). For visceral pain, psychological distress plays a more integral role in both development and amplification than for somatic pain (Sikandar and Dickenson 2012), making psychological domain assessment arguably even more important in this context.

The relevance of specific domains depends on clinical context, stakeholder perspectives, assessment timeframe, and feasibility, all of which vary across settings.

The first step in developing a COS is to map what has already been measured in existing trials (Williamson et al. 2017). This systematic review therefore aims to map and critically evaluate the domains, outcome measures, and contextual factors used to assess pain in RCTs of acute abdominal pain, serving as a foundation for the possible future development of a COS for acute abdominal pain.

## 2 Methods

This systematic review was conducted in accordance with PRISMA 2020 guidelines (Page et al. 2021) and was prospectively registered in the International Prospective Register of Systematic Reviews (PROSPERO; ID: CRD42025630171). No protocol beyond registration was prepared.

### 2.1 Eligibility Criteria

We included RCTs that investigated patients experiencing acute abdominal (visceral) pain of any cause, excluding studies focused solely on postoperative pain or acute pancreatitis, which are covered by separate reviews within our research group (Prospero; ID: CRD420251121922 (Joseph et al. 2025)). Eligible studies had to report pain assessment methods as part of the trial methodology. Data extraction focused on domains (based on recommendations from the COMET initiative (Williamson et al. 2017)) and measures, both unidimensional (e.g., Visual Analogue Scale [VAS], Numeric Rating Scale [NRS]) and multidimensional (e.g., McGill Pain Questionnaire), regardless of whether they are validated or exploratory, related to acute abdominal pain. Only studies published in English from 1 January 2000, up to 3 February 2025, were considered. Studies were excluded if they were non‐RCTs, non‐human studies, studies focused exclusively on chronic pain (three months or longer), or if data on acute pain or mixed populations could not be extracted separately.

### 2.2 Information Sources and Search Strategy

A comprehensive search was conducted in PubMed, Embase, Scopus, and Web of Science on 3 February 2025. The final search strategy used a combination of keywords and Boolean operators: (‘acute pain’) AND (‘visceral pain’ OR ‘abdominal pain’) AND (‘pain assessment’ OR ‘pain measurement’) AND (‘randomized controlled trial’ OR ‘RCT’). Filters were applied to limit results to English‐language RCTs published from 1 January 2000, to the date of the final search. Reference lists of included studies and relevant reviews were also screened to undertake a reverse citation searching, but yielded no additional eligible publications.

All records from the searches were imported into Covidence, a web‐based platform for systematic review management (Veritas Health Innovation). Title and abstract screening were performed independently by two reviewers (LK, CSK). Disagreements were resolved through discussion. Articles deemed potentially relevant were retrieved in full text and assessed independently by the same two reviewers according to predefined eligibility criteria. If disagreements persisted, a third reviewer (SP) was consulted to reach a consensus. In cases of multiple publications from the same study, the most comprehensive or recent report was included, or multiple publications were merged if they provided additional pain assessment methods.

### 2.3 Data Extraction

Data extraction was initiated using a standardized extraction form within Covidence, adapted to the present review. Two reviewers independently extracted each article. The following data were collected:

- Study characteristics: publication year, country, study population, and intervention type.
- Pain‐related outcome domains: domains named by the author and/or based on specific measures, including Patient Reported Outcome Measures (PROMs); these PROMs were categorized and aggregated under their domains as recommended by COMET (Williamson et al. 2017).
- Pain‐related outcome measures:
  ○ Type of measure (PROM, Patient‐reported Experience Measure [PREM], report, etc.)
    - For PROMs/PREMs: tool name, type (unidimensional/multidimensional), domains assessed (intensity, quality, etc.), and rationale for use (if reported), self‐report vs. observer‐based; delivery format (e.g., paper‐based, electronic, verbal).
- Context of use: inpatient vs. outpatient; type of intervention (pharmacological, procedural, etc.).
- Frequency of use and diagnostic specificity of each tool.
- Ability to detect change over time (e.g., pre‐ and post‐intervention scores).
- Compliance and feasibility metrics, where available.
- Reporting quality: clarity, consistency, and alignment with standardized guidelines (e.g., CONSORT).

### 2.4 Inter‐Rater Agreement

To ensure consistency and reliability in screening and extraction, all steps were performed in duplicate. Interrater agreement was assessed during the full‐text review.

### 2.5 Data Synthesis and Comparison

Assessment tools were mapped and summarized using narrative synthesis and descriptive statistics. Tools were classified by type (unidimensional vs. multidimensional), the domains assessed, and their frequency of use across included studies.

Temporal trends in assessment frequency were examined using linear regression with publication year as the predictor and number of repeated measurements as the outcome. Robust standard errors were applied.

The risk of bias for each included study was evaluated using the Cochrane Risk of Bias 2 tool, which assesses five domains: bias arising from the randomization process, bias due to deviations from intended interventions, bias due to missing outcome data, bias in the measurement of outcomes, and bias in the selection of the reported result (Sterne et al. 2019). Each domain was rated as low risk of bias, some concerns, or high risk of bias, and an overall judgement was derived for each study. The assessment was performed independently by two reviewers (LK and CSK), and discrepancies were resolved through discussion.

Following peer review, we additionally classified each trial post hoc according to the diagnostic status of the population at enrolment, as either undifferentiated acute abdominal pain or a defined condition, based on the reported inclusion criteria. Outcome domains, measurement instruments, and assessment schedules were then compared between these groups. This analysis was exploratory, not pre‐specified in the PROSPERO registration, and is reported descriptively without statistical testing.

## 3 Results

A total of 826 studies were identified through database searches (PubMed *n* = 258; Embase *n* = 243; Scopus *n* = 195; Web of Science *n* = 130). After the removal of 381 duplicates, 445 unique records were screened by title and abstract. Eighty full‐text articles were assessed for eligibility, of which 35 were excluded: 19 lacked pain‐related outcomes, 13 included ineligible patient populations, and 3 were not randomized designs (Figure 1). The studies that did not include pain‐related outcomes either investigated conditions where pain was only one component of the symptom profile or focused on diagnostic accuracy following analgesic treatment.

**FIGURE 1:**
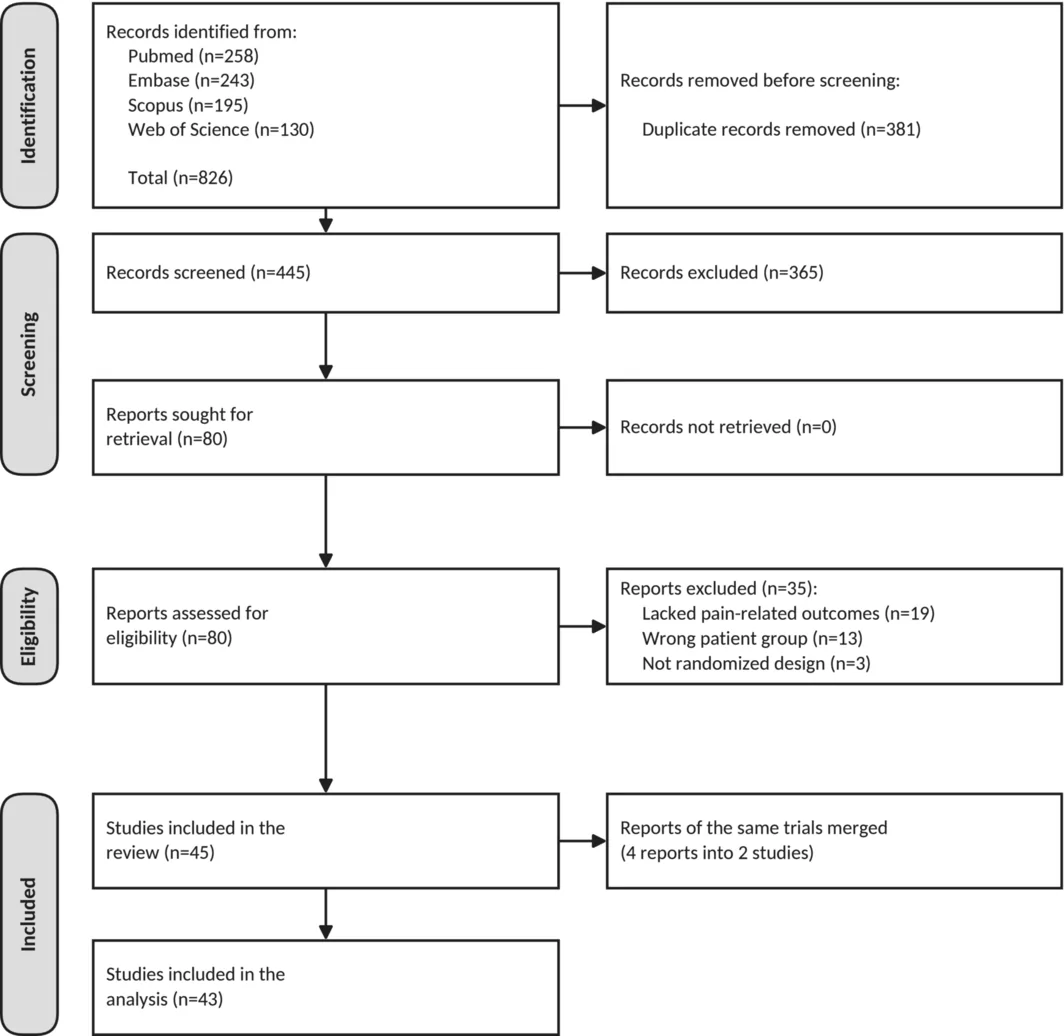
PRISMA flowchart. PRISMA flow diagram of the study identification, screening, eligibility assessment, and inclusion process.

Two pairs of publications reported data from the same cohorts but provided different pain assessment data and were therefore merged in the analysis (Abril Ochoa et al. 2020; Bailey, Bergeron, Gravel, and Daoust 2007; Bailey, Bergeron, Gravel, Bussières, and Bensoussan 2007; Chinn et al. 2019). The final sample included 43 RCTs, comprising 5284 patients from 15 countries across four continents. Most studies were conducted in North America (*n* = 23), followed by Asia/Middle East (*n* = 10), Europe (*n* = 9), and Africa (*n* = 1).

### 3.1 Study Characteristics

Study characteristics are summarized in Table 1. All studies included patients presenting with acute abdominal pain at the emergency department. Most studies included adults (*n* = 35/43); three of these also included participants aged < 18 years (ranging from 12 years and up, ensuring that all patients were able to read and answer relevant questions). One study included only patients aged > 65 years.

**TABLE 1:** Study characteristics.

Author	Publication year	Country	Intervention	Sample size	Study design	Pain outcomes
Adult studies						
D. Aghamo‐hammadi	2012	Iran	Morphine	120	Parallel group, double‐blinded	Opioid treatment did not affect diagnostic accuracy
O. Agodirin	2013	Nigeria	Tramadol	95	Parallel group, double‐blinded	Tramadol treatment did not affect diagnostic accuracy
J. Antevil	2005	USA	Glycopyrrolate	39	Parallel group, double‐blinded	No improvement in pain of suspected biliary tract origin in treatment with glycopyrrolate
J. Bartfield	2003	USA	Intravenous fentanyl vs. Nebulised fentanyl	50	Parallel group, double‐blinded	Nebulised fentanyl provided comparable analgesia to intravenous fentanyl
A. Birnbaum	2012	USA	Patient‐controlled analgesia (morphine)	211	Parallel‐group, open‐label	Patient‐controlled analgesia provides high treatment satisfaction and more effective analgesia than traditional treatment
A. Chang	2013	USA	Hydromorphone vs. Standard care	350	Parallel group, double‐blinded	2 mg hydromorphone resulted in a higher proportion of patients declining further analgesic treatment than standard treatment after 30 min
E. Chinn	2019	USA	Lidocaine vs. hydromorphone	154	Parallel group, double‐blinded	Intravenous hydromorphone was superior to intravenous lidocaine for general abdominal pain
T. Deaton	2015	USA	IV morphine vs. nebulised fentanyl	40	Parallel group, double‐blinded	Fentanyl citrate at a dose of 2 μg/kg through a breath‐actuated nebulizer appears to be a feasible and safe alternative to intravenous morphine in patients with acute abdominal pain
J. Gallagher	2006	USA	Morphine	160	Parallel group, double‐blinded	Opioid treatment did not affect diagnostic accuracy
A. Giacosa	2022	Italy	*Boswellia serrata*	49	Parallel group, double‐blinded	*Boswellia Serrata* provides pain relief beyond placebo in patients with acute diarrhoea
K. Kanesan	2023	Malaysia	Patient‐controlled analgesia vs. bolus (opioids)	62	Parallel group, open‐label	There was no significant difference in pain score reduction between PCA and intravenous bolus of morphine for the management of severe acute non‐traumatic abdominal pain in the ED. However, PCA provided more patient satisfaction and should be considered as an alternative modality of acute pain management in the ED
K. Kang	2015	Korea	Morphine	219	Parallel group, double‐blinded	Early pain control did not affect diagnostic accuracy
W. Kruis	2013	Germany	Mesalazine	123	Parallel group, double‐blinded	A daily dose of 3.0 g mesalazine may relieve pain during a symptomatic flare of uncomplicated diverticulitis
S. Lovett	2021	USA	Low vs. high dose ketamine	98	Parallel group, double‐blinded	No considerable difference in analgesia or adverse effect profile between 0.15 mg/kg IV ketamine and 0.30 mg/kg was detected in the short‐term treatment of acute pain in the ED
M. Mahadevan	2000	Singapore	Tramadol	68	Parallel group, blinded; however, blinding was not described in detail	Opioid treatment did not affect diagnostic accuracy
C. Maity	2019	India	Lactobacillus	60	Parallel group, double‐blinded	*Bacillus coagulans* was effective in recovering from acute diarrhoea with abdominal pain and discomfort
B. McMahon	2024	USA	TENS	81	Parallel‐group, sham‐controlled	TENS to the abdominal wall did not provide efficient pain relief in abdominal pain
J. Miller	2015	USA	Morphine vs. low‐dose ketamine	45	Parallel group, double‐blinded	Low‐dose ketamine did not produce a greater reduction in NRS pain scores compared with morphine for acute pain in the emergency department
S. Motov (1)	2015	USA	Ketamine vs. morphine	90	Parallel group, double‐blinded	Subdissociative intravenous ketamine provides comparable analgesia to intravenous morphine
S. Motov (2)	2017	USA	IV push vs. infusion of low‐dose ketamine	48	Parallel group, double‐blinded	Low‐dose ketamine given as a short infusion is associated with significantly lower rates of feeling of unreality and sedation, with no difference in analgesic efficacy in comparison to intravenous push
S. Motov (3)	2017	USA	Ketolac	240	Parallel group, double‐blinded	Ketorolac has similar analgesic efficacy at intravenous doses of 10, 15, and 30 mg
S. Motov (4)	2019	USA	Ketamine vs. morphine	60	Parallel group, double‐blinded	Subdissociative ketamine provides analgesic efficacy comparable to morphine for short‐term treatment of acute pain in the geriatric ED patients, but results in higher rates of psychoperceptual adverse effects
S. Motov (5)	2019	USA	Ibuprofen	225	Parallel group, double‐blinded	Oral ibuprofen administered at doses of 400, 600, and 800 mg has similar analgesic efficacy for short‐term pain relief in adult patients presenting to the ED with acute pain
M. Moussa	2021	Lebanon	Diclofenac	150	Parallel group, double‐blinded	Intradermal sterile water injection and diclofenac were equally effective for the pain relief of acute renal colic
J. Mozafari	2020	Iran	Ketamine vs. fentanyl	130	Parallel group, double‐blinded	Ketamine was found to be less effective than fentanyl in controlling renal colic‐induced pain and to be associated with a higher prevalence of side effects
S. Narang	2018	India	Drotaverine + paracetamol vs. paracetamol alone	252	Parallel group, double‐blinded	Fixed‐dose combination of drotaverine hydrochloride (80 mg) and PCM (500 mg) is an effective and safe antispasmodic agent in abdominal pain associated with acute infectious gastroenteritis
T. Nguyen	2024	USA	IV vs. nebulised ketamine	150	Parallel group, double‐blinded	No difference between the administration of IV and nebulised ketamine for the short‐term treatment of moderate to severe acute pain in the ED
H. Oguzturk	2012	Turkey	Tramadol/paracetamol	210	Parallel group, double‐blinded	Early administration of tramadol and paracetamol provided effective pain relief in patients with non‐traumatic acute abdominal pain, and the administration did not interfere with diagnosis
V. Ojetti	2022	Italy	Probiotics	119	Parallel group, double‐blinded	*L. reuteri* 4659 did not give substantial pain relief in patients with uncomplicated diverticulitis
C. Petruzziello	2019	Italy	Probiotics ( *Lactobacillus reuteri* )	88	Parallel group, double‐blinded	*Lactobacillus reuteri* reduced abdominal pain in patients with uncomplicated diverticulitis
J. Remington‐Hobbs	2012	United Kingdom	Hyoscine/hyoscine + paracetamol/paracetamol	132	Parallel group, double‐blinded	Oral paracetamol is at least as effective as intravenous hyoscine butylbromide and a combination of both drugs in the management of acute undifferentiated abdominal pain presenting to the ED
B. Sin	2019	USA	Morphine vs. sufentanil	60	Parallel group, double‐blinded	The use of i.n. Sufentanil resulted in rapid and safe analgesia with comparable efficacy to i.v. Morphine for up to 30 min in patients who presented with acute pain in the ED
S. Thomas	2003	USA	Morphine	74	Parallel group, double‐blinded	Opioid treatment did not affect diagnostic accuracy
C. Yildirim	2024	Turkey	Hyoscine‐n‐butylbromide	50	Parallel group, double‐blinded	No clinical or statistical difference in analgesic effect
C. Zhu	2017	China	Hyoscine butylbromide vs. anisodamine	299	Parallel group, double‐blinded	Hyoscine butylbromide was non‐inferior to anisodamine in pain relief of patients with acute gastric or intestinal spasm‐like pain
Paediatric studies						
B. Bailey	2007	Canada	Morphine	90	Parallel group, double‐blinded	Only the visual analogue scale and colour analogue scale had a limit of agreement less than the limit of acceptability of ±20 mm
G. Cozzi	2019	Italy	Ketorolac/tramadol/paracetamol	210	Parallel group, patients were blinded, but physicians were not	Similar analgesic effect in all groups
R. Green	2005	Canada	Morphine	108	Parallel group, double‐blinded	Opioid treatment did not affect diagnostic accuracy
J. Kang	2021	China	Modified endoscopic retrograde appendicitis therapy vs. antibiotics	94	Parallel group, open label	Modified Endoscopic Retrograde Appendicitis Therapy provides a new alternative therapeutic option for children with uncomplicated acute appendicitis, especially for families who are reluctant to undergo an appendectomy
M. Kim	2002	USA	Morphine	60	Parallel group, double‐blinded	Opioid treatment did not affect diagnostic accuracy
H. Kokki	2005	Finland	Oxycodone	63	Parallel group, double‐blinded	Early administration of buccal oxycodone provides significant pain relief in children with acute abdominal pain
N. Poonai	2020	Canada	Hyoscine	236	Parallel group, double‐blinded	Hyoscine butylbromide provided equal pain relief as acetaminophen in children with acute pain
K. Quinn	2018	USA	Ketamine vs. fentanyl	22	Parallel group, double‐blinded	Intranasal ketamine was found to be inferior to intranasal fentanyl in relieving pain at 10 min and was found to have significantly greater rates of sedation and dizziness

Eight of 43 studies focused exclusively on paediatric populations with an age range of 1–18 years.

Sample sizes ranged from 39 to 350 participants (median = 98) in adult studies and 22–236 participants in paediatric studies (median 92). Inclusion and exclusion criteria are summarized in Table S1.

All eligible studies were interventional, enrolling patients directly from Emergency Departments. Interventions in the adult studies primarily involved analgesic treatments (*n* = 29/35) (Aghamohammadi et al. 2012; Agodirin et al. 2013; Antevil et al. 2005; Bartfield et al. 2003; Birnbaum et al. 2012; Chang et al. 2013; Chinn et al. 2019; Deaton et al. 2015; Gallagher et al. 2006; Kanesan and Rahman 2023; Kang et al. 2015; Lovett et al. 2021; Mahadevan and Graff 2000; Miller et al. 2015; Motov et al. 2015; Motov, Mai, et al. 2017; Motov, Yasavolian, et al. 2017; Motov, Masoudi, et al. 2019; Motov, Mann, et al. 2019; Moussa et al. 2021; Mozafari et al. 2020; Narang and Koli 2018; Nguyen et al. 2024; Oguzturk et al. 2012; Remington‐Hobbs et al. 2012; Sin et al. 2019; Thomas et al. 2003; Yildirim and Pamukçu Günaydin 2024; Zhu et al. 2017), procedural interventions (*n* = 1/35) (McMahon et al. 2024), or disease‐specific medical treatments (*n* = 5/35) (Giacosa et al. 2022; Kruis et al. 2013; Maity and Gupta 2019; Ojetti et al. 2022; Petruzziello et al. 2019). Analgesic interventions included antispasmodics, non‐opioid analgesics, ketamine, and opioids. The procedural intervention was transcutaneous electrical nerve stimulation. In paediatric studies, interventions included analgesic treatment (*n* = 7/8) (Bailey, Bergeron, Gravel, and Daoust 2007; Bailey, Bergeron, Gravel, Bussières, and Bensoussan 2007; Cozzi et al. 2019; Green et al. 2005; Kim et al. 2002; Kokki et al. 2005; Poonai et al. 2020; Quinn et al. 2018) and procedural interventions (*n* = 1/8) (Kang et al. 2021). Analgesic treatments were similar in paediatric and adult studies. The sole intervention was endoscopic treatment of appendicitis.

All included studies used pain assessment methods, and the different pain domains assessed are summarized in Table 2 and Figure 2.

**TABLE 2:** Assessment of pain domains^a^.

Author	Pain intensity	Frequency	Pain relief	Rescue analgesic/total dose	Adverse effects	Pattern	Duration	Location	Physical examination	Quality of life
Adult studies										
D. Aghamo‐hammadi	Numeric Rating Scale 0–10	At baseline and after 25 min			No side effects were reported				Abdominal tenderness, abdominal rebound	
O. Agodirin	Numeric Rating Scale 0–10	At baseline and after 15–20 min			Not reported					
J. Antevil	Visual Analogue Scale 0–100 mm	At baseline and after 20 min							Rebound tenderness, involuntary guarding, and pain migration	
J. Bartfield	Visual Analogue Scale 0–100 mm	At baseline, after 15 and 30 min								
A. Birnbaum	Visual Analogue Scale 0–10	At baseline, after 30, 60, 90, and 120 min	Patient satisfaction	Total dose	Sedation, hypotension, oxygen desaturation					
A. Chang	Numeric Rating Scale 0–10	At baseline, after 15, 30, 45, 60, 90, and 120 min	Declined further analgesics, achieved > 50% reduction in NRS, achieved NRS ≤ 3 in 30 min, complete resolution of pain in 30 min		Bradycardia, hypotension, pruritus, nausea, vomiting					
E. Chinn	Numeric Rating Scale 0–10	At baseline, after 15, 30, 45, 60, 90, and 120 min	Patient satisfaction	Rescue analgesics	Dizziness, drowsiness, headache, nausea, pruritus		Yes			
T. Deaton	Visual Analogue Scale 0–100 mm	At baseline, after 10, 20, 30, and 40 min	Patient satisfaction, physician satisfaction	Rescue analgesics						
J. Gallagher	Visual Analogue Scale 0–100 mm	At baseline and after 15 min								
A. Giacosa	Visual Analogue Scale 0–10	At baseline and at day 6							Abdominal distension	
K. Kanesan	Visual Analogue Scale 0–10	At baseline, after 30, 60, and 120 min	Experience of pain relief Patient satisfaction	Total dose	No significant adverse effects were reported					
K. Kang	Visual Analogue Scale 0–10 cm	At baseline and after 30 min			Not reported					
W. Kruis	Visual Analogue Scale 0–100 mm, A 4‐point pain intensity Likert scale (no, slight, moderate, severe, most severe)	At baseline and daily for 6 weeks	4‐point treatment efficacy Likert scale (ineffective, moderately effective, effective, very effective)		Headache, diarrhoea	Pain frequency 0–100 mm	Yes			
S. Lovett	Numeric Rating Scale 0–10	At baseline, after 15, 30, and 60 min	Patient satisfaction	Total dose, rescue analgesics	Fatigue, dizziness, headache, unreal feeling, hearing changes, vision changes, mood alterations, discomfort, hallucination		Yes			
M. Mahadevan	Visual Analogue Scale 0–100 mm	At baseline, after 30, and 60 min						Yes	Percussion tenderness, light/deep tenderness, rebound tenderness, cough tenderness	
C. Maity	Universal Pain Assessment Scale (mild, moderate, severe)	At baseline, after 24, 48, and 120 h			Not reported					Not reported
B. McMahon	Numeric Rating Scale 0–10	At baseline and after 30 min		Analgesics prior to admission, rescue analgesics	No adverse effects of treatment reported			Yes		
J. Miller	Numeric Rating Scale 0–10	At baseline, after 15, 30, 45, 60, 75, 90, 105, and 120 min	Patient satisfaction	Total dose	Nausea, vomiting, oxygen de‐saturation, hallucination					
S. Motov (1)	Numeric Rating Scale 0–10	At baseline, after 5 min, after 10, 20, 40, 60, 80, 100, and 120 min	Complete resolution of pain, reduction of NRS ≥ 3	Rescue analgesics	Dis‐orientation, dizziness, mood changes, nausea					
S. Motov (2)	Numeric Rating Scale 0–10	At baseline, after 15, 30, 60, 90, and 120 min		Rescue analgesics	Fatigue, dizziness, headache, unreal feeling, vision changes, mood alterations, discomfort, hallucination					
S. Motov (3)	Numeric Rating Scale 0–10	At baseline, after 5, 15, 30, 60, 90, and 120 min		Rescue analgesics	Dizziness, nausea, headache, pruritus, flushing					
S. Motov (4)	Numeric Rating Scale 0–10	At baseline, after 15, 30, 60, 90, and 120 min	Complete resolution of pain, reduction of pain by NRS ≥ 3	Rescue analgesics	Fatigue, dizziness, headache, unreal feeling, hearing changes, vision changes, mood alterations, discomfort, hallucination					
S. Motov (5)	Numeric Rating Scale 0–10	At baseline and after 60 min		Rescue analgesics	No adverse effects reported					
M. Moussa	Visual Analogue Scale 0–10 cm	At baseline, after 30, 45, and 60 min		Rescue analgesics	Nausea, vomiting, epigastric pain, and pain at the injection site					
J. Mozafari	Visual Analogue Scale 0–10 cm	At baseline, after 5, 15, and 30 min		Rescue analgesics	Nausea, vomiting, headache, dizziness, agitation					
S. Narang	Visual Analogue Scale 0–100 mm, A 4‐point pain intensity Likert scale	At baseline, after 15, 30, 60, 90, and 120 min, after 24, 48, and 72 h	Global improvement score (patient), global improvement score (physician)		Dizziness, vomiting, nausea, headache, weakness, sleepiness, periorbital burning, body ache	Pain pattern				
T. Nguyen	Numeric Rating Scale 0–10	At baseline, after 15, 30, 60, 90, and 120 min		Rescue analgesics	Sedation, restlessness, dizziness, an unreal feeling					
H. Oguzturk	Visual Analogue Scale 0–100 mm	At baseline, after 20 and 40 min			Nausea, vomiting, dizziness				Rebound tenderness, rigidity	
V. Ojetti	Visual Analogue Scale 0–10 cm	At baseline and daily for 10 days			Not reported					
C. Petruzziello	Visual Analogue Scale 0–10 cm	At baseline and daily for 10 days			No adverse effects reported					
J. Remington‐Hobbs	Visual Analogue Scale 0–100 mm	At baseline, after 30, 60, and 180 min		Rescue analgesics	Blurred vision, dizziness, drowsiness, dry mouth, palpitations, spaced out, nausea					
B. Sin	Numeric Rating Scale 0–10	At baseline, after 5, 10, 20, and 30 min	Patient satisfaction	Rescue analgesics	Nausea, vomiting, dizziness, drowsiness, oxygen desaturation					
S. Thomas	Visual Analogue Scale 0–10 cm	At baseline and after 60 min						Yes	Signs of peritoneal irritation	
C. Yildirim	Visual Analogue Scale 0–100 mm	At baseline, after 30, and 60 min		Rescue analgesics	Dizziness					
C. Zhu	Numeric Rating Scale 0–10	At baseline, after 10, 20, 30, 60, and 120 min	Patient satisfaction	Rescue analgesics	Thirst, dry mouth, nodal arrhythmia, hernia, eventration, and ileus					
Paediatric studies										
B. Bailey	Visual Analogue Scale 0–100 mm, Colour Analogue Scale, Wong‐Baker FACES, Numeric Rating Scale	At baseline and after 30 min		Rescue analgesics	Pruritus, nausea, vomiting, drowsiness, dizziness		Yes	Yes	Involuntary guarding, rebound tenderness	
G. Cozzi	Numeric Rating Scale 0–10, Wong Baker FACES	At baseline, after 30, 60, and 120 min			Nausea, vomiting, dizziness, epigastric pain					
R. Green	The Colour Analogue Scale	At baseline and after 15 min			Not reported				Right lower quadrant tenderness, involuntary/voluntary guarding	
J. Kang		At the end of the study	Symptom resolution		Not reported		Yes			
M. Kim	Visual Analogue Scale 0–10	At baseline and after 30 min			Nausea, pruritus				Palpatory tenderness area, percussion tenderness area	
H. Kokki	Visual Analogue Scale 0–10 cm	At baseline and every 30 min for 3.5 h			Headache, urticaria				Abdominal guarding, right lower quadrant pain, migration of pain, rebound tenderness	
N. Poonai	Visual Analogue Scale 0–100 mm	At baseline, after 15, 30, 45, 60, and 80 min		Rescue analgesics	Nausea, dizziness, dry mouth, photo‐sensitivity, vomiting, constipation, dry skin, palpitations, diarrhoea, sweating, drowsiness, headache, urticaria					
K. Quinn	Numeric Rating Scale 0–10, Wong Baker FACES	At baseline, after 10, 20, 30, and 60 min		Analgesics before enrolment, rescue analgesics	Dizziness, sedation					

^a^ As no studies examined psychological features or pain interference, this part has been left out of the table.

**FIGURE 2:**
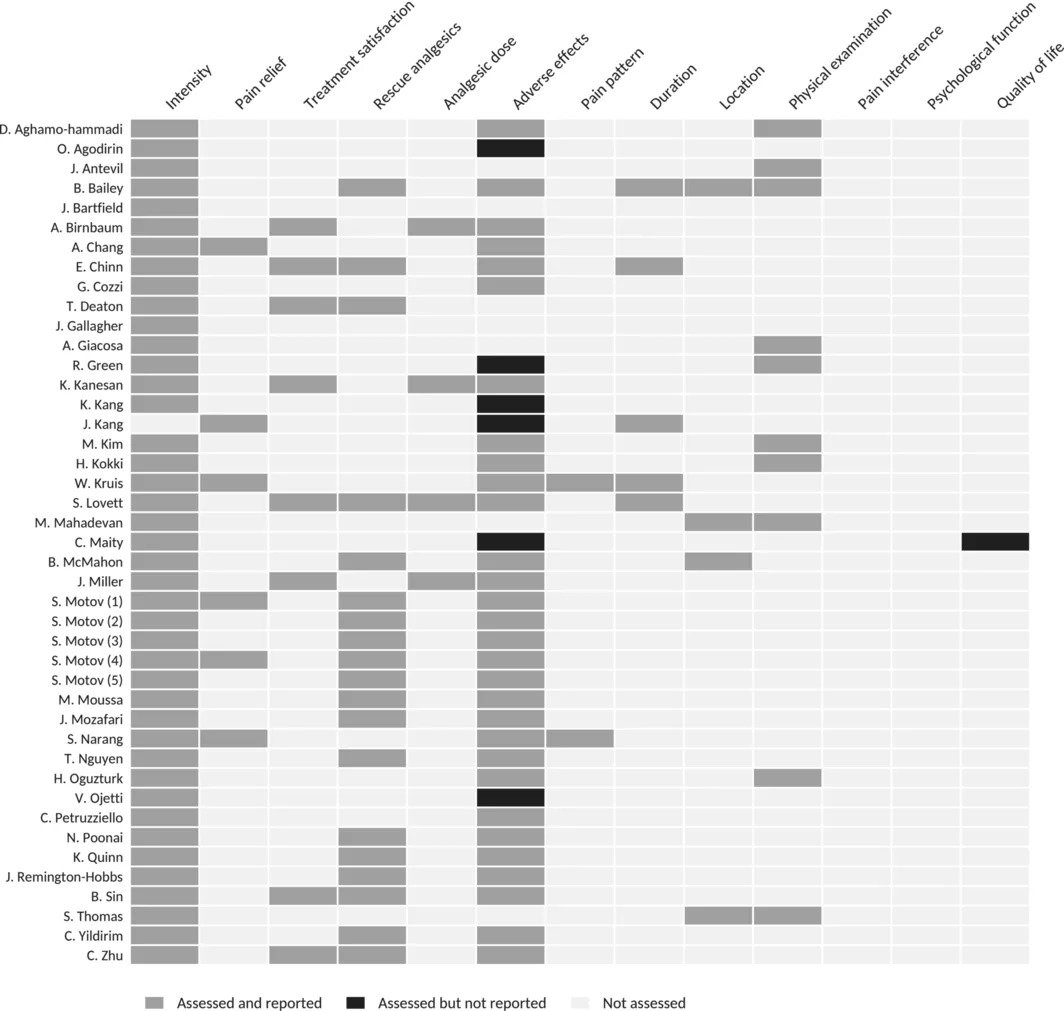
Heatmap over assessed pain domains. Heatmap illustrating the distribution of pain‐related outcome domains assessed across included randomized controlled trials. Rows represent individual studies, columns represent predefined pain‐related domains, and shading indicates assessment and reporting. Light grey, not assessed; Grey, assessed and reported; Black, assessed, but not reported.

Trend analysis suggested an increase in assessment frequency over time (*β* = 0.11 per year, *p* = 0.032) when excluding an extreme outlier with daily measurements for > 1 month, indicating that more recent studies tend to include a higher number of repeated pain measurements. Studies published after 2013 typically reported 4–7 repeated assessments, whereas studies from the early 2000s commonly included only 2–3 (Figure 3).

**FIGURE 3:**
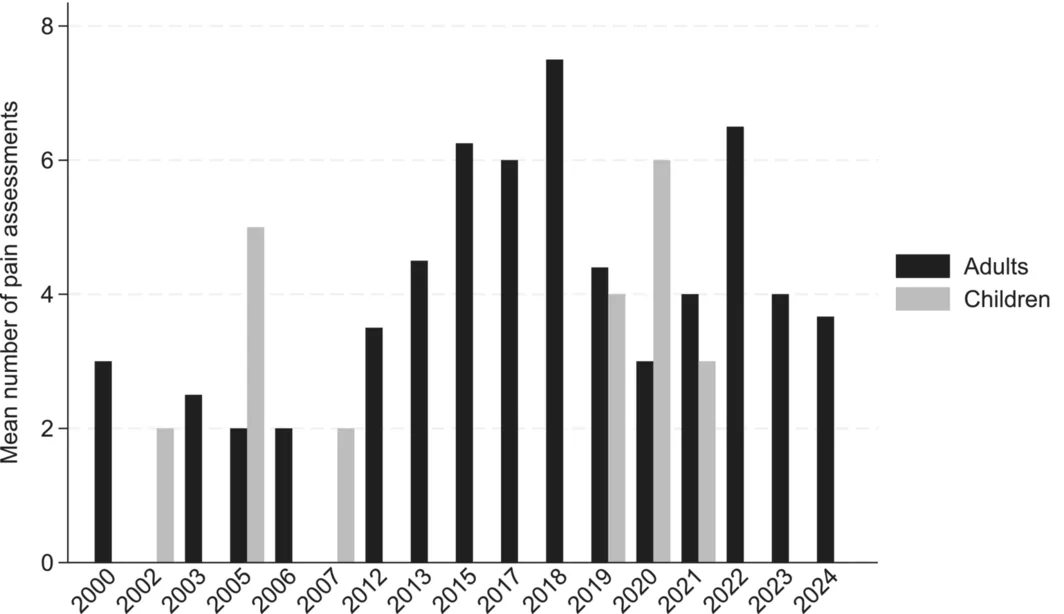
Temporal trend in number of pain assessments throughout the studies. Temporal trends in the mean number of pain assessments reported per trial over time, stratified by adult and paediatric studies. Bars represent the mean number of pain assessments per study for each publication year. One extreme outlier (Kruis et al. 2013), which included daily pain measurements over 6 weeks (> 40 timepoints), was excluded to avoid distortions of the overall trend.

### 3.2 Outcome Domains According to the Underlying Condition

Twenty‐nine of the 43 (67%) trials enrolled patients with undifferentiated acute abdominal pain, recruited on the basis of the symptom and its severity rather than an established diagnosis, while 14 (33%) trials enrolled patients with a defined condition: acute diarrhoea or gastroenteritis (*n* = 4), suspected or confirmed appendicitis (*n* = 4), acute uncomplicated diverticulitis (*n* = 3), renal colic (*n* = 2), and suspected biliary disease (*n* = 1). The classification of each trial is reported in Table S2. Ten of the trials classified as undifferentiated recruited patients with acute pain at mixed anatomical sites, with abdominal pain as one of several eligible locations. Pain intensity was assessed in 42 of the 43 (98%) trials across all diagnostic groups, the exception of a paediatric trial of endoscopic treatment for appendicitis that reported symptom resolution only. Analgesic use was reported in 18 of 29 (62%) trials of undifferentiated pain compared with 4 of 14 (29%) condition‐specific trials, and pain relief or treatment satisfaction in 11 of 29 (40%) compared with 3 of 14 (21%). Of the 10 trials that recorded physical examination findings, 9 (90%) enrolled patients in whom a surgical diagnosis was under consideration, and the temporal pattern of pain was assessed only in one trial of diverticulitis and one of infectious diarrhoea (20%). The assessment schedule separated the groups more clearly than the domains did, in that trials of undifferentiated pain followed patients over minutes to two hours after a single analgesic intervention, whereas trials in diverticulitis and acute diarrhoea followed pain daily over days to weeks. The NRS was the primary instrument in 17 of 29 (59%) trials of undifferentiated pain and in none of the condition‐specific trials, which used the VAS or a verbal Likert scale. No trial in any diagnostic group assessed pain interference, emotional distress or quality of life.

### 3.3 Adult Studies

#### 3.3.1 Number of Pain‐Related Domains

Most adult trials assessed three pain‐related domains (*n* = 15/35), while only 12 studies out of 35 assessed more than three domains (Figure 4). Over time, there has been a shift from one single domain (pain intensity) towards multi‐domain capture (e.g., pain relief, rescue analgesia, adverse effects) (*β* = 0.05 per year, *p* = 0.048).

**FIGURE 4:**
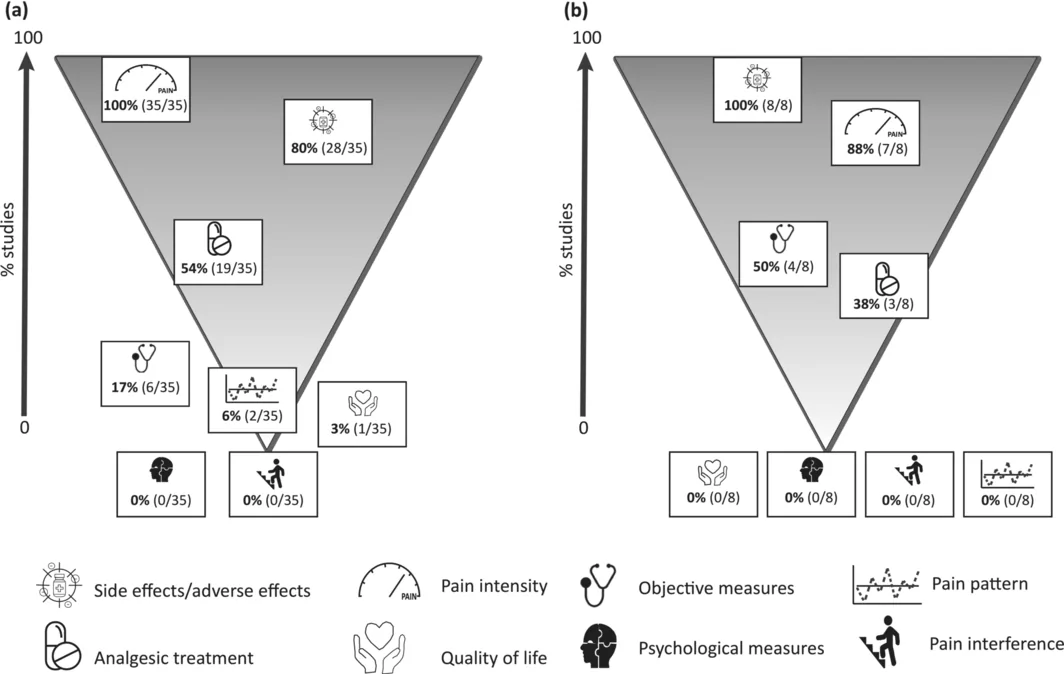
Occurrence of the domains assessed in studies on acute abdominal pain. Distribution of pain‐related outcome domains assessed in randomized controlled trials of acute pancreatitis, shown separately for adult (a) and paediatric (b) studies. The funnels depict the proportion of studies reporting each domain; numbers in parentheses indicate the number of studies.

#### 3.3.2 Pain Intensity Domain

All adult studies reported pain intensity outcomes. The most frequently used instrument to assess pain intensity was the VAS (*n* = 19, 54%) (Antevil et al. 2005; Bartfield et al. 2003; Birnbaum et al. 2012; Deaton et al. 2015; Gallagher et al. 2006; Giacosa et al. 2022; Kanesan and Rahman 2023; Kang et al. 2015; Kruis et al. 2013; Mahadevan and Graff 2000; Moussa et al. 2021; Mozafari et al. 2020; Narang and Koli 2018; Oguzturk et al. 2012; Ojetti et al. 2022; Petruzziello et al. 2019; Remington‐Hobbs et al. 2012; Thomas et al. 2003; Yildirim and Pamukçu Günaydin 2024), followed by the NRS (*n* = 15, 43%) (Aghamohammadi et al. 2012; Agodirin et al. 2013; Chang et al. 2013; Chinn et al. 2019; Lovett et al. 2021; McMahon et al. 2024; Miller et al. 2015; Motov et al. 2015; Motov, Mai, et al. 2017; Motov, Yasavolian, et al. 2017; Motov, Masoudi, et al. 2019; Motov, Mann, et al. 2019; Nguyen et al. 2024; Sin et al. 2019; Zhu et al. 2017). Three studies out of 35 used Likert scales, where two employed both VAS and a Likert scale (Kruis et al. 2013; Narang and Koli 2018), and one study used only a Likert scale (Maity and Gupta 2019).

All studies evaluated pain intensity at rest; none during activity.

In 83% of adult studies (*n* = 29/35), pain intensity was assessed within the first two hours after treatment (Aghamohammadi et al. 2012; Agodirin et al. 2013; Antevil et al. 2005; Bartfield et al. 2003; Birnbaum et al. 2012; Chang et al. 2013; Chinn et al. 2019; Deaton et al. 2015; Gallagher et al. 2006; Kanesan and Rahman 2023; Kang et al. 2015; Lovett et al. 2021; Mahadevan and Graff 2000; Maity and Gupta 2019; McMahon et al. 2024; Miller et al. 2015; Motov et al. 2015; Motov, Mai, et al. 2017; Motov, Yasavolian, et al. 2017; Motov, Masoudi, et al. 2019; Motov, Mann, et al. 2019; Moussa et al. 2021; Mozafari et al. 2020; Nguyen et al. 2024; Oguzturk et al. 2012; Sin et al. 2019; Thomas et al. 2003; Yildirim and Pamukçu Günaydin 2024; Zhu et al. 2017), while 6 out of 35 assessed pain for ≥ 72 h (Giacosa et al. 2022; Kruis et al. 2013; Maity and Gupta 2019; Narang and Koli 2018; Ojetti et al. 2022; Petruzziello et al. 2019). Long‐duration follow‐ups employed Likert scales (*n* = 1, 3%) (Maity and Gupta 2019), VAS (*n* = 3, 9%) (Giacosa et al. 2022; Ojetti et al. 2022; Petruzziello et al. 2019), or both (*n* = 2, 6%) (Kruis et al. 2013; Narang and Koli 2018).

Pain intensity was assessed a median of four times per study, with Likert scales being used more frequently than NRS/VAS (mean = 18.7 vs. 4.7 assessments).

A temporal trend for the use of pain intensity measurement tools was observed: prior to 2010, VAS was predominant (VAS = 8; NRS = 1 of 9), while from approximately 2011 onward, NRS use gradually increased and became dominant in studies published after 2015 (NRS = 10; VAS = 9 of 20) (Figure 5).

**FIGURE 5:**
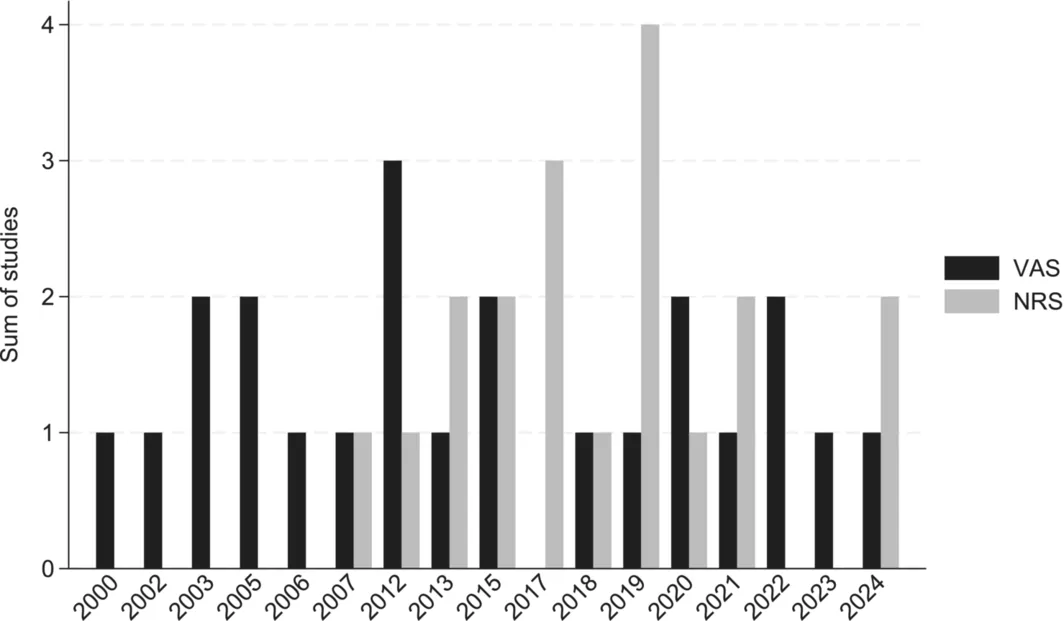
Temporal trend in pain intensity assessment. Use of unidimensional pain intensity scales over time in randomized controlled trials. Bars show the number of studies per publication year reporting Numeric Rating Scale (NRS) or Visual Analogue Scale (VAS) assessments.

#### 3.3.3 Analgesic Domain

##### 3.3.3.1 Rescue Analgesics and Total Analgesic Use

Nineteen studies assessed analgesic use. Four of 35 studies reported the mean dose of analgesics administered (Birnbaum et al. 2012; Kanesan and Rahman 2023; Lovett et al. 2021; Miller et al. 2015); 16 reported the use of rescue analgesia (Chinn et al. 2019; Deaton et al. 2015; Lovett et al. 2021; McMahon et al. 2024; Motov et al. 2015; Motov, Mai, et al. 2017; Motov, Yasavolian, et al. 2017; Motov, Masoudi, et al. 2019; Motov, Mann, et al. 2019; Moussa et al. 2021; Mozafari et al. 2020; Nguyen et al. 2024; Remington‐Hobbs et al. 2012; Sin et al. 2019; Yildirim and Pamukçu Günaydin 2024; Zhu et al. 2017).

Twenty‐three of 35 adult trials excluded patients who had received analgesics prior to enrollment: 16 excluded all pre‐treatment analgesics, and 7 excluded only opioid use. Of the remaining 12 studies, only one reported whether pre‐inclusion analgesia had been administered (McMahon et al. 2024).

##### 3.3.3.2 Treatment Satisfaction

Eight studies reported treatment satisfaction (Birnbaum et al. 2012; Chinn et al. 2019; Deaton et al. 2015; Kanesan and Rahman 2023; Lovett et al. 2021; Miller et al. 2015; Sin et al. 2019; Zhu et al. 2017). One study additionally evaluated physician satisfaction (Deaton et al. 2015). Not all studies provided information on the method of assessment; some asked whether patients were satisfied with the treatment effect, and some asked whether patients would prefer the same analgesic treatment for future reference. No study reported the use of validated instruments or questions for this assessment.

##### 3.3.3.3 Adverse Drug Effects

28/35 studies assessed treatment side effects or adverse effects (Aghamohammadi et al. 2012; Agodirin et al. 2013; Birnbaum et al. 2012; Chang et al. 2013; Chinn et al. 2019; Kanesan and Rahman 2023; Kang et al. 2015; Kruis et al. 2013; Lovett et al. 2021; Maity and Gupta 2019; McMahon et al. 2024; Miller et al. 2015; Motov et al. 2015; Motov, Mai, et al. 2017; Motov, Yasavolian, et al. 2017; Motov, Masoudi, et al. 2019; Motov, Mann, et al. 2019; Moussa et al. 2021; Mozafari et al. 2020; Narang and Koli 2018; Nguyen et al. 2024; Oguzturk et al. 2012; Ojetti et al. 2022; Petruzziello et al. 2019; Remington‐Hobbs et al. 2012; Sin et al. 2019; Yildirim and Pamukçu Günaydin 2024; Zhu et al. 2017), four studies claimed to assess side effects or adverse effects, but did not report either in the result section (Agodirin et al. 2013; Kang et al. 2015; Maity and Gupta 2019; Ojetti et al. 2022). Studies typically either used open‐ended questions or did not specify the method for assessing side effects. No measurement instrument for adverse events/side effects was mentioned in any of the studies.

#### 3.3.4 Pain Pattern, Duration, and Location Domains

Two adult studies assessed pain pattern (constant vs. intermittent or frequency of pain) through a pain diary (Kruis et al. 2013; Narang and Koli 2018), and three reported pain duration or time to resolution (Chinn et al. 2019; Kruis et al. 2013; Lovett et al. 2021) but did not specify how the duration was assessed. Three adult studies (Mahadevan and Graff 2000; McMahon et al. 2024; Thomas et al. 2003) reported pain location.

#### 3.3.5 Physical Examination Domain

Six of the 35 adult studies (Aghamohammadi et al. 2012; Antevil et al. 2005; Giacosa et al. 2022; Mahadevan and Graff 2000; Oguzturk et al. 2012; Thomas et al. 2003) recorded objective findings, including rebound tenderness, voluntary or involuntary guarding, cough tenderness, and abdominal distension. The findings were typically predefined, but no measurement instrument was mentioned.

#### 3.3.6 Pain Interference, Psychological Outcomes, and Quality of Life Domains

No study assessed how pain interfered with physical function (e.g., mobilization). Quality of life was listed as an outcome in one adult study, but the assessment method was not clearly defined. From the results section, it was indicated that the quality of life was based on the presence of constipation, diarrhoea, or blood/mucus in stool, rather than functional effects (Maity and Gupta 2019). No study reported psychological outcomes.

### 3.4 Paediatric Studies

#### 3.4.1 Number of Pain‐Related Domains

Paediatric studies evaluated 2–6 domains. No temporal trend in domains assessed was seen in paediatric studies (*β* = −0.05 per year, *p* = 0.232).

#### 3.4.2 Pain Intensity Domain

Among paediatric studies, all but one study reported pain intensity (*n* = 7/8); the remaining study reported only pain duration and resolution (Kang et al. 2021). The studies generally used age‐appropriate instruments such as the Wong–Baker FACES Pain Scale or the Colour Analogue Scale (*n* = 4/8) (Bailey, Bergeron, Gravel, and Daoust 2007; Cozzi et al. 2019; Green et al. 2005; Quinn et al. 2018). One paediatric trial compared four different intensity tools (VAS, FACES, NRS, and Colour Analogue Scale) (Bailey, Bergeron, Gravel, and Daoust 2007). Three studies used only the VAS, and the lowest inclusion age in these studies was 4 years of age (Kim et al. 2002; Kokki et al. 2005; Poonai et al. 2020).

All studies assessed pain intensity at rest.

In the paediatric studies, 5 out of 8 studies assessed pain within the first two hours after treatment and no studies had long‐duration follow‐ups beyond 72 h.

#### 3.4.3 Analgesic Domain

##### 3.4.3.1 Rescue Analgesics and Total Analgesic Use

No studies reported the mean dose of analgesics administered; three of eight studies reported use of rescue analgesics (Bailey, Bergeron, Gravel, Bussières, and Bensoussan 2007; Poonai et al. 2020; Quinn et al. 2018).

Five of the studies excluded patients who had received analgesics prior to enrollment, where two excluded all analgesics and three only excluded opioid treatment. One of the remaining three studies reported whether analgesia had been administered before inclusion.

##### 3.4.3.2 Adverse Drug Effects

All studies assessed treatment side effects and/or adverse effects, but two studies did not report the results (Green et al. 2005; Kang et al. 2021). No paediatric study reported treatment satisfaction.

#### 3.4.4 Pain Pattern, Duration, and Location Domains

Two paediatric studies reported pain duration without specifying the mode of assessment (Bailey, Bergeron, Gravel, Bussières, and Bensoussan 2007; Kang et al. 2021). No study reported pain pattern. One paediatric study (Bailey, Bergeron, Gravel, Bussières, and Bensoussan 2007) reported pain location from a standardized objective abdominal assessment rather than patient report.

#### 3.4.5 Physical Examination Domain

Four studies (Bailey, Bergeron, Gravel, Bussières, and Bensoussan 2007; Green et al. 2005; Kim et al. 2002; Kokki et al. 2005) recorded objective findings similar to the findings reported in adult studies.

#### 3.4.6 Pain Interference, Psychological Outcomes, and Quality of Life Domains

Pain interference, psychological function, and quality of life were not assessed in any of the paediatric studies.

### 3.5 Risk of Bias

Risk of bias was evaluated using the Cochrane Risk of Bias 2 tool. Most studies were judged to have low risk of bias in domains related to randomization, deviations from intended interventions, and outcome measurement (Table 3). However, concerns were frequent for missing outcome data and especially for selective reporting, where most studies were rated as having *some concerns*.

**TABLE 3:** Risk of bias assessment.

Author	Bias from the randomization process	Bias due to deviations from intended interventions	Bias due to missing outcome data	Bias in the measurement of the outcomes	Bias in the selection of the reported result	Overall risk of bias
Adult studies						
D. Aghamohammadi	Low	Low	Low	Low	Some concerns	Some concerns
O. Agodirin	Low	Low	Low	Low	High	High
J. Antevil	Low	Low	Low	Low	Some concerns	Some concerns
J. Bartfield	Low	Low	Low	Some concerns	Low	Some concerns
A. Birnbaum	Low	Low	Low	Some concerns	Low	Some concerns
A. Chang	Low	Low	Low	Some concerns	Low	Some concerns
E. Chinn	Low	Low	Low	Low	Low	Low
T. Deaton	Low	Low	Low	Low	Some concerns	Some concerns
J. Gallagher	Low	Low	Low	Low	Some concerns	Some concerns
A. Giacosa	Some concerns	Low	Low	Low	Low	Some concerns
K. Kanesan	Low	Low	Low	Some concerns	Some concerns	Some concerns
K. Kang	Low	Low	Low	Low	High	High
W. Kruis	Low	Low	Low	Some concerns	Low	Some concerns
S. Lovett	Low	Low	Low	Low	Low	Low
M. Mahadevan	Low	Low	Low	Low	Some concerns	Some concerns
C. Maity	Low	Low	Low	Low	High	High
B. McMahon	Low	Low	Low	Low	Some concerns	Some concerns
J. Miller	Some concerns	Low	Low	Low	Some concerns	Some concerns
S. Motov (1)	Low	Low	Low	Low	Low	Low
S. Motov (2)	Low	Low	Low	Low	Low	Low
S. Motov (3)	Low	Low	Low	Low	Some concerns	Some concerns
S. Motov (4)	Low	Low	Low	Low	Low	Low
S. Motov (5)	Low	Low	Low	Low	Some concerns	Some concerns
M. Moussa	Low	Low	Low	Low	Some concerns	Some concerns
J. Mozafari	Low	Low	Low	Low	Some concerns	Some concerns
S. Narang	Low	Low	Low	Low	Some concerns	Some concerns
T. Nguyen	Low	Low	Low	Low	Low	Low
H. Oguzturk	Low	Low	Low	Low	Some concerns	Some concerns
V. Ojetti	Low	Low	Low	Low	High	High
C. Petruzziello	Low	Low	Low	Low	Some concerns	Some concerns
J. Remington‐Hobbs	Low	Low	Low	Low	Some concerns	Some concerns
B. Sin	Low	Low	Low	Low	Low	Low
S. Thomas	Low	Low	Low	Low	Some concerns	Some concerns
C. Yildirim	Low	Low	Low	Low	Low	Low
C. Zhu	Low	Low	Low	Low	Low	Low
Paediatric studies						
B. Bailey	Low	Low	Low	Low	Some concerns	Some concerns
G. Cozzi	Low	Low	Low	Some concerns	Low	Some concerns
R. Green	Low	Low	Low	Low	High	High
J. Kang	Low	Low	Low	Low	High	High
M. Kim	Low	Low	Low	Low	Some concerns	Some concerns
H. Kokki	Low	Low	Low	Low	Some concerns	Some concerns
N. Poonai	Low	Low	Low	Low	Low	Low
K. Quinn	Low	Low	Low	Low	Low	Low

Common issues included the absence of trial preregistration and unblinded study designs. Four of 35 adult studies were rated as high risk of bias in one or more domains (Agodirin et al. 2013; Kang et al. 2015; Maity and Gupta 2019; Ojetti et al. 2022), primarily due to missing reports of predefined outcomes. Twenty‐two studies had some concerns in one or more domains (Aghamohammadi et al. 2012; Antevil et al. 2005; Bartfield et al. 2003; Birnbaum et al. 2012; Chang et al. 2013; Deaton et al. 2015; Gallagher et al. 2006; Giacosa et al. 2022; Kanesan and Rahman 2023; Kruis et al. 2013; Mahadevan and Graff 2000; McMahon et al. 2024; Miller et al. 2015; Motov, Yasavolian, et al. 2017; Motov, Masoudi, et al. 2019; Moussa et al. 2021; Mozafari et al. 2020; Narang and Koli 2018; Oguzturk et al. 2012; Petruzziello et al. 2019; Remington‐Hobbs et al. 2012; Thomas et al. 2003).

Two of the eight paediatric studies were rated as high risk of bias in one or more domains (Green et al. 2005; Kang et al. 2021), primarily due to missing reports of predefined outcomes. Four studies had some concerns in one or more domains (Bailey, Bergeron, Gravel, Bussières, and Bensoussan 2007; Cozzi et al. 2019; Kim et al. 2002; Kokki et al. 2005).

Overall, the evidence base for evaluating current practice in acute abdominal pain assessment was considered to be of acceptable methodological quality, with predominantly low to moderate risk of bias.

Studies with a lower risk of bias more frequently included clinically relevant effect measures such as patient satisfaction and responder‐based definitions of pain relief. In contrast, high‐risk of bias studies rarely assessed pain relief, and instead tended to include nonspecific objective abdominal signs (e.g., guarding, rebound tenderness) which were not captured in low‐risk studies.

## 4 Discussion

This systematic review identified 43 randomized controlled trials including 5284 patients with acute abdominal pain. Of these, eight studies (883 patients) focused solely on paediatric patients. Almost all studies assessed pain intensity, most commonly using the Visual Analogue Scale or the Numeric Rating Scale. Over time, a gradual shift was observed from VAS to NRS as the preferred tool for evaluating pain intensity, and there was a trend towards more frequent measures. Pain was typically assessed within the first two hours after treatment, and only a minority of studies reported pain beyond 72 h. Measures of pain interference, psychological domains, or quality of life were entirely omitted. Also, multidimensional assessment methods were virtually never used. Although most studies demonstrated low to moderate risk of bias, selective reporting, and lack of preregistration were frequent concerns.

### 4.1 Interpretation and Implications

The findings of the review suggest that research related to interventions for acute abdominal pain has predominantly focused on unidimensional pain intensity measures. This focus is not unexpected, given the acute setting and short follow‐up periods of most included trials. However, while such tools are simple, quick, and familiar to clinicians, they capture only one aspect of the pain experience (Crisman et al. 2024). Acute abdominal pain is a complex experience that includes sensory, psychological, cognitive, and autonomic factors (Kuner and Kuner 2021). Limiting assessment to intensity alone may therefore restrict interpretability of treatment effects and mask clinically meaningful differences in patient outcomes, particularly in studies aiming to evaluate outcomes beyond immediate analgesic relief (Crombez et al. 2023).

These tools were not specifically validated for visceral pain or in the context of acute abdominal pain (Hjermstad et al. 2011). The near absence of multidimensional tools (Crisman et al. 2024), such as the McGill Pain Questionnaire (Melzack 1975) or the Brief Pain Inventory (MD Anderson Cancer Center), suggests that clinical trials in this field continue to rely on a relatively narrow assessment of pain.

The INTEGRATE‐pain initiative recommends inclusion of pain (intensity and interference), physical function, and quality of life as core outcome domains in acute pain trials (Bova et al. 2023). Similarly, IMMPACT and PedIMMPACT recommendations propose broader domain coverage, including pain relief, onset and duration of analgesic effects, patient satisfaction, adverse effects, and emotional response (Cooper et al. 2016; McGrath et al. 2008). Few of the included studies incorporated these domains, reflecting a gap between recommended and applied outcome assessment.

Outcome assessment in acute abdominal pain is substantially less comprehensive than in chronic abdominal pain research, particularly chronic pancreatitis, where pain characteristics, psychological function, and quality of life are routinely considered (Teo et al. 2016).

The appropriateness of pain domains might depend on the clinical context and time frame of assessment. In emergency department settings with short observation periods, rapid and simple measures such as pain intensity and analgesic use may be most feasible to capture immediate treatment effects. However, if the situation of a patient is stabilized, domains such as physical functioning, quality of life, and psychological function should still be assessed in the acute phase, as recommended for acute pain in general (Bova et al. 2023).

Although paediatric trials tended to include slightly more pain‐related domains than adult trials, they still relied almost exclusively on pain intensity scales and short‐term outcomes. Despite well‐recognized developmental differences in pain communication, no study incorporated paediatric PROMs beyond pain intensity (e.g., functional interference or emotional distress).

The trials differed in whether a diagnosis had been established at enrolment, and this was reflected in what was measured. In undifferentiated abdominal pain, where the trials asked whether a single analgesic dose relieves pain in the emergency department, the domains assessed were pain intensity, analgesic use, pain relief, and adverse effects, all captured within two hours. In trials of a defined condition such as diverticulitis or infectious diarrhoea, where treatment targets the disease rather than the pain, pain intensity was followed daily for days or weeks alongside the temporal pattern of symptoms. Physical examination findings were recorded almost exclusively in trials where a surgical diagnosis was under consideration, which reflects the longstanding concern that analgesia masks diagnostic signs rather than an interest in pain experience. The domains shared across conditions were therefore those tied to the immediate treatment of pain, whereas the underlying condition determined the timeframe of follow‐up and whether examination findings were recorded.

Future trials should adopt a context‐specific approach to outcome selection, where domains are aligned with both the clinical purpose and the time frame of assessment.

To address the identified gaps, a Core Outcome Set (COS) for acute abdominal pain should be developed to establish which domains and measures should be consistently assessed across trials. This process should engage all relevant stakeholders—including clinicians, researchers, regulators, and patient representatives—to ensure that the selected outcomes reflect both scientific rigour and patient priorities.

### 4.2 Temporal Trends and Clinical Considerations

The observed shift from VAS to NRS over the past decade mirrors developments in other pain settings. NRS is generally preferred for its simplicity, ease of administration, and lower cognitive demand (Firdous et al. 2021; Hjermstad et al. 2011). One study in this review compared the psychometric properties of scales in children and found that the VAS and NRS had wide limits of agreement, thereby precluding their use interchangeably (Bailey, Bergeron, Gravel, and Daoust 2007). A previous review comparing VAS and NRS, however, showed that the two correlate, except for a few studies where VAS scores were systematically higher than NRS scores (Hjermstad et al. 2011).

Most studies assessed pain only during the initial hours after treatment, reflecting a focus on immediate analgesic efficacy rather than sustained relief, side effects, functional, and psychological recovery. The lack of long‐term follow‐up limits understanding of whether early pain reduction translates into better patient outcomes, such as earlier mobilization, greater diagnostic certainty, or improved satisfaction (Cooper et al. 2016).

These observations should be interpreted with caution, as we did not account for factors that may influence the choice of outcome measure and assessment frequency, including study rationale and design.

### 4.3 Strengths and Limitations

This review followed PRISMA 2020 guidelines, used duplicate screening and extraction, and applied a validated risk‐of‐bias tool. Inclusion was limited to RCTs, increasing methodological robustness. However, potential limitations include restriction to English‐language publications and possible publication bias towards positive findings. Because the included trials varied widely in diagnosis, setting, timing of pain assessment, and choice of pain measures, statistical pooling was not feasible. We therefore relied on descriptive synthesis, although this approach may mask more subtle patterns in how pain is assessed across studies.

Our comparison of outcome domains according to the underlying condition was exploratory and performed post hoc. The condition‐specific groups were small, ranging from one to four trials, and diagnosis was closely tied to intervention type, follow‐up duration, and publication year, so the differences we describe cannot be attributed to the underlying condition alone. A further source of heterogeneity is that 10 of the included trials recruited patients with acute pain at mixed anatomical sites, with abdominal pain as one of several eligible locations, so their outcome assessment was not designed for abdominal pain specifically.

The gaps identified here resemble those in other acute pain settings, such as acute postoperative pain (Bigalke et al. 2021; Maeßen et al. 2021), where the need for a COS has led to the recent development of a COS of domains (Pogatzki‐Zahn et al. 2021) and, respectively, PROMs (Pogatzki‐Zahn et al. 2025).

Measurement instruments used in trials and clinical practice for abdominal pain–specific assessments should be chosen based on COSMIN recommendations, including construct validity and responsiveness in this unique pain setting (Bova et al. 2023; Cooper et al. 2016; Williamson et al. 2017).

According to COSMIN guidance, content validity is the most important measurement property and should be demonstrated before evaluating other properties such as reliability or responsiveness (Gagnier et al. 2021).

Taken together, our findings highlight a misalignment between what is commonly measured in acute abdominal pain trials and what patients, clinicians, guidelines, and regulators increasingly consider to be necessary for a more comprehensive assessment of pain and what constitutes meaningful changes. At the moment, a COS for acute pain should be used to assess the most relevant outcomes in clinical acute abdominal pain trials and clinical practice (Bova et al. 2023). However, because abdominal pain is a particular entity, the development of a core outcome set specific to different types of acute abdominal pain would promote standardization across trials and strengthen the interpretability of future evidence.

## Author Contributions

L.K., N.J., S.S.O., J.W., A.M.D., S.P., and E.P.‐Z. designed the study. L.K. and C.S.K. performed the data extraction; S.P. and N.J. were available for resolving any disagreements. L.K. and E.P.‐Z. analysed the data. L.K. had a primary role in preparing the manuscript, which E.P.‐Z., A.M.D., and S.P. edited. All authors have approved the final version of the manuscript and agree to be accountable for all aspects of the work.

## Funding

The authors have nothing to report.

## Conflicts of Interest

The authors declare no conflicts of interest.

## Supporting information


**Table S1:** Study inclusion and exclusion criteria.
**Table S2:** Classification of included trials according to the population enrolled.
