# Supplementary material for: Pain‐Related Outcome Domains and Measures in Randomized Controlled Trials of Treatment for Acute Abdominal Pain: A Systematic Review
Source: Eur J Pain. 2026 Nov 4;30(10):e70445. doi: 10.1002/ejp.70445 (PMC13732789; doi:10.1002/ejp.70445)
Supplement: Supplementary file 1 [file EJP-30-0-s001.docx]

**Table S1: Study inclusion and exclusion criteria**

|  | Author | Inclusion criteria | Exclusion criteria |
| --- | --- | --- | --- |
|  | *Adult studies* | | |
|  | D. Aghamo-hammadi | Acute surgical abdomen patients who were scheduled for urgent abdominal surgery and were ready to be transferred to the operating room | People with an addiction, patients younger than 12 years old, and patients who have received intravenous opioids or oral pain killers within the last week. |
|  | O. Agodirin | Acute abdomen, age 18-60 | Abdominal symptoms more than 7 days before presentation, hypotensive or obtunded patients, inability to comprehend the pain scoring method, reaction to tramadol or opiates, and prior treatment with analgesics in the course of the illness. |
|  | J. Antevil | Age 18-65, acute right upper quadrant pain or tenderness, or persistent epigastric pain, not relieved by standard treatment (mixture of lidocaine and antacid) | Active upper gastrointestinal haemorrhage, haematuria, pregnancy, choledocholithiasis, acute pancreatitis, history of coronary artery disease, congestive heart  Failure, arrhythmias, valvular heart disease,  acute coronary syndrome, altered sensorium, systolic blood pressure >200 mm Hg or diastolic >115 mm Hg, glaucoma or myasthenia gravis, allergy to anticholinergic medications, peritonitis |
|  | J. Bartfield | Adult patients (≥18 years), abdominal pain requiring intravenous fentanyl as part of their treatment | Allergy to fentanyl or other opioids, inability to give informed consent, or judged to be in extremis. |
|  | A. Birnbaum | Age 18-65,  abdominal pain of at most 7 days' duration,  and deemed by the ED attending physician to require IV  opioid analgesia | Use of opioids within 24 hours, long-term use of opioids, or chronic pain syndrome, clinician's suspicion of opioid  dependence or abuse, clinical suspicion of intoxication, pregnancy or breastfeeding, chronic obstructive pulmonary disease, sleep apnoea, renal insufficiency, or renal failure, oxygen saturation of < 97% on room air or systolic blood pressure (sBP) < 100 mm Hg, use of monoamine oxidase inhibitors, phenothiazines, or tricyclic antidepressants; prior allergic reaction to morphine; inability to provide informed  consent, previous entry into the study. |
|  | A. Chang | 21-64 years, acute pain if pain was of sufficient severity to warrant use of IV opioids in the judgment of the EP | Patients managed by any of the EPs who were investigators in this study, allergy to hydromorphone or morphine, saturation < 95%, alcohol or other drug, use of other opioids within the past 7 days, use of a monoamine oxidase inhibitor, pregnancy, chronic pain, or weight less than 150 pounds. |
|  | E. Chinn | Aged 18-64, weight 60-120 kg, acute, severe abdominal pain warranting opioid treatment for no more than 7 days. Patients who received off-protocol medication in the ED before enrolment were eligible for enrolment if greater than 1 hour had elapsed since off-protocol medication administration and the patient still met inclusion criteria (pain warranting intravenous opioids) | Cardiac conduction system impairment, renal or liver disease, hemodynamic instability, pregnancy, breastfeeding, allergy to either medication, reported use of opioids within the previous week, chronic pain disorder |
|  | T. Deaton | Age 18-65, acute noninjury abdominal pain, pain ≥5 out of 10, pain less than 24-hour duration, no allergy to morphine sulphate or fentanyl citrate,  able to provide informed consent, not determined to be in extremis. | Impairment in renal or hepatic function, hypothyroidism, Addison's disease, prostatic hypertrophy, urethral stricture, pregnancy, lactation, treatment with monoamine oxidase inhibitors, tricyclic antidepressants, sedative hypnotics, cytochrome P450 3A4 inhibitors within 14 days before enrolment, or patients who had taken oral or had been administered IV or intramuscular pain medications before enrolment |
|  | J. Gallagher | Age 21 years or older, atraumatic abdominal pain of less than 48-hour duration, judged by the ED attending physician to warrant opioid analgesia for pain control | Isolated flank pain, pregnancy, morphine allergy, analgesic treatment before screening, concurrent painful sickle cell crisis, hypotension, inability to provide informed consent |
|  | A. Giacosa | Diagnosis of acute diarrhoea with at least three liquid stools pr day for no more than 5 days, age 18–70 | Long-term diarrhoea, a concomitant illness that could cause diarrhoea, iatrogenic diarrhoea, disease requiring hospitalisation and intravenous treatment, high temperature (>38.5 ◦C), subjects treated by other anti-diarrheal and antibiotic drugs in the previous 24 h, severely malnourished subjects, pregnancy |
|  | K. Kanesan | Adult patients, severe non-traumatic acute (less than 24 hours of onset) abdominal pain with a VAS score at triage of more than seven | Previous history of chronic pain of any cause, Glasgow Coma Scale of less than 15, hypotension, breathing rate <10/min, dementia or acute confusion states, visual impairment and blindness, allergy, inability to gain intravenous access, reduced pulmonary capacity, prior treatment of acute pain of different modalities |
|  | K. Kang | Suspected acute appendicitis, ≥15 years, nontraumatic right lower quadrant pain of less than 48 hours' duration | Pregnancy, patients transferred from other hospitals with a diagnosis of appendicitis, received analgesics for abdominal pain, renal insufficiency, or allergy to computed tomography (CT) contrast |
|  | W. Kruis | Age 45–80 years, diverticulitis with acute pain without serious complications, lower abdominal pain of moderate or severe intensity on at least four of the previous 7 days, a minimum of four diverticula observed on endoscopy examination at baseline, at least four of eight specified symptoms present for at least the previous 2 days and still present at study inclusion (i.e. abdominal pain localised mainly in the lower left part of the abdomen; abdominal pain enhanced after meals; abdominal pain decreased after defaecation or wind; bloating; constipation defined as two defaecations/week; diarrhoea, defined as >3 loose stools per day; a sensation of incomplete evacuation after defaecation; and painful lower left abdomen at palpation) | Chronic inflammatory bowel disease,  fever or other signs of serious complications,  severe renal disease, defined as serum creatinine  >1.5 mg/dL, known intolerance to the study medication, and a requirement for prohibited concomitant medication. |
|  | S. Lovett | Age 18-59, acute flank, abdominal, back, musculoskeletal, or headache pain, intensity ≥5 (NRS), requiring intravenous (IV) analgesics. Acute pain was defined as onset within 7 days or less. | Pregnancy, breastfeeding, weight <45 or >115 kg, altered mental status, known allergy to ketamine, history of chronic pain, currently taking opioid medication, renal dysfunction, or hepatic insufficiency, history of a seizure, traumatic head or eye injury, unstable vital signs, active alcohol or drug use disorder, acute alcohol or drug intoxication, or acute psychiatric illness |
|  | M. Mahadevan | >11 years, right lower quadrant pain for less than a week's duration (nontraumatic in origin), suggestive of acute appendicitis | None described |
|  | C. Maity | 18–65 years, acute diarrhoea manifesting within 48 h, at least three incidences of unformed stool, last stool type 7 by Bristol Stool form scale within 24h, abdominal discomfort within the last hour | Severe form diarrhoea or diarrhoea as symptoms of more severe disease complexities, purulent or bloody stools, require antibiotic treatment, and the presence of ulcers, pregnancy, disease emergencies, concomitant therapy, |
|  | B. McMahon | Adult patients, moderate to severe abdominal pain, verbal numeric pain score ≥5 | Inability to verbally communicate, inability to consent, allergy to analgesics, history of peptic ulcer or gastrointestinal bleeding, clinical instability, pregnancy, dermatologic condition that precludes application of TENS pads, pacemaker, patient refusal, and provider refusal. |
|  | J. Miller | Age 18-59 years, abdominal, flank, low back, or extremity pain that the ED provider felt warranted IV opioid treatment | Pathological vital signs, altered mental status, intoxication, opioids or tramadol use, ischemic heart disease, heart failure or unstable dysrhythmias, use of an opioid or tramadol within 4 hours prior to enrolment, morphine or ketamine allergies, required pain medication immediately, pregnant or breastfeeding, oxygen-dependent pulmonary disease, hepatic cirrhosis, or dialysis, presence of intracranial mass, a history of psychosis, weight less than 45 kg or greater than 115 kg, or presence of acute ocular or head trauma. |
|  | S. Motov (1) | Age 18 to 55, acute abdominal, flank, back, or musculoskeletal pain (onset within 7 days), pain score ≥5, opioid requiring | Pregnancy, breastfeeding, weight less than 46 kg or greater than 115 kg, unstable vitals, medical history of acute head or eye injury, seizure, intracranial hypertension, chronic pain, renal or hepatic insufficiency, alcohol or drug abuse, psychiatric illness, or recent opioid use (4 hours) |
|  | S. Motov (2) | Adults aged 18–65 with acute abdominal, flank, back, chest, or musculoskeletal pain and NRS ≥ 5. Awake, alert, able to provide consent and rate pain. | Pregnancy, breastfeeding, altered mental status, ketamine allergy, weight <46 or >115 kg, unstable vitals, seizure, head/eye trauma, renal/hepatic insufficiency, substance use disorder, psychiatric illness, or recent analgesic use. |
|  | S. Motov (3) | Age 18–65, acute pain (≤30 days), NRS ≥5, ketorolac deemed appropriate | Age >65, renal/hepatic disease, GI bleed, NSAID allergy, abnormal vitals, recent analgesic use, pregnancy |
|  | S. Motov (4) | Aged ≥65 years, acute pain (≤7 days), pain NRS ≥5, abdominal/flank/back/MSK pain, deemed to require opioid. | Altered mental status, allergy to morphine or ketamine, weight below 40 kg or above 115 kg, unstable vital signs, and past medical history of acute head or eye injury, seizure, intracranial hypertension, severe chronic obstructive pulmonary disease, chronic pain, renal or hepatic insufficiency, alcohol or drug abuse, psychiatric illness, or recent opioid use. |
|  | S. Motov (5) | Adults (≥18 yrs), acute pain needing oral ibuprofen as judged by the physician. | Peptic ulcer disease, GI bleed, renal/hepatic insufficiency, NSAID allergy, altered mental status, opioid/NSAID use within 4 hrs, pregnancy/lactation. |
|  | M. Moussa | Age 18–60, acute renal colic diagnosed by CT | Age <18 or >60, prior analgesics, local infection at the injection site |
|  | J. Mozafari | Age 15–65, symptoms consistent with renal colic, confirmed by ultrasound or CT | Psychiatric illness, opioid use, pregnancy, significant comorbidities (renal, liver, CV, CNS), allergies, recent analgesic use, etc. |
|  | S. Narang | 18-59 years,  acute infectious diarrhoea, moderate-to-severe abdominal pain | Severe dehydration, GI bleeding, chronic GI conditions, serious comorbidities, pregnancy/lactation, medications with GI side effects |
|  | T. Nguyen | ≥18 years, acute painful conditions, initial pain score ≥ 5, deemed appropriate to receive ketamine analgesia, awake, alert, and oriented | Immediate need for treatment, unstable vitals, intoxication, ketamine allergy, BMI >150 kg, altered mental status, pregnancy, substance abuse |
|  | H. Oguzturk | >17 years, abdominal pain less than 72 hours' duration, no traumatic origin | Pregnancy, allergy to opioids or paracetamol, hypotension, self-medication with analgesia |
|  | V. Ojetti | Age 18–80 years, fever < 38°C, CRP < 180 mg/L, diagnosis of acute uncomplicated diverticulitis (Hinchey = 0), CT-confirmed | Fever > 38°C, CRP > 180 mg/L, prior colonic surgery, IBD, cancer, pregnancy/lactation, antibiotics or probiotics in the prior 7–15 days, major systemic disease, mental illness |
|  | C. Petruzziello | Age >18, fever >38°C, CT-confirmed acute uncomplicated diverticulitis (Hinchey = 0), informed consent | <18 years, prior colonic surgery, pregnancy/lactation, recent use of antibiotics or probiotics, IBD, major systemic diseases, contrast/antibiotic allergies, psychiatric illness |
|  | J. Remington-Hobbs | Adults with abdominal pain | Age <65, age <17, duration >7 days, obvious gynaecological disorder, peritonism, haemodynamic instability, pregnancy, paracetamol within the last 4 hours, medicated before screening |
|  | B. Sin | ≥18 years, English-speaking, NRS ≥ 4, acute pain ≤15 days | Hemodynamic instability, AMS, pregnancy, recent analgesia, nasal obstruction, chronic disease (e.g., renal/liver failure), trauma/headache |
|  | S. Thomas | Adults ≥18 years, non-pregnant, undifferentiated abdominal pain <72 hrs, severe enough to consider opioid use, hemodynamically stable. | Pregnant, hypotensive, known morphine allergy, requiring urgent/emergent treatment or obvious indication for early opioid use (e.g., renal colic), non-severe pain. |
|  | C. Yildirim | Age 18-65, at least three watery stools in the last 24 hours, duration of watery stool 2 weeks or less, abdominal pain | Unable to use the visual analogue scale (VAS), analgesic drugs within four h before the ED visit, hemodynamic abnormalities (systolic blood pressure of <100 mmHg, heart rate of >100/ min), peritonitis/acute abdomen, allergy to the study drugs, diabetes mellitus or neuropathic diseases, any medications in the ED before being included in the study, pregnancy, gastroenteritis not likely diagnosis |
|  | C. Zhu | Age 18–70, acute gastric or intestinal spasm-like pain (NRS ≥6), no organic cause requiring intervention, presenting to ED. | Organic abdominal diseases needing intervention (e.g., perforation, obstruction), analgesic or GI-active meds in the past seven half-lives, malignancy, or psychiatric illness. |
|  | *Pediatric studies* | | |
|  | B. Bailey | Age 8-18, pain in the right lower quadrant of the abdomen, pain of 3 days or fewer, NRS ≥5, a presumptive diagnosis of appendicitis requiring a  surgical consultation | Appendicitis proven by ultrasonography or computed  tomography (CT) before surgical assessment, analgesia other than acetaminophen or ibuprofen given before enrolment, hemodynamically unstable, sepsis, immunocompromised, sickle cell anaemia, previous abdominal surgery, inflammatory bowel disease, pancreatic or biliary disease, allergy to morphine, and suspected or present pregnancy. |
| G. Cozzi | | Age 4-18 years of age, moderate to  severe acute abdominal pain for <48 hours, self-reported pain of at least six on an appropriate rating scale for their age. | Allergy or sensitivity to NSAIDs, opioids, or paracetamol, use of analgesic drugs in the last 8 hours, nephropathy, liver disease, metabolic or neurologic disease, and thrombocytopenia or bleeding disorders, suspected faecal stasis or severe dehydration |
| R. Green | | Age 5-16, nontraumatic abdominal pain less than 48-hour duration, a surgical consultation was warranted for a possible surgical condition. | Allergy to opiates, opiate use within the past 4 hours, hypotension, absence of parents |
| J. Kang | | 1-13 years, diagnosis of acute uncomplicated appendicitis, family's refusal to have a standard surgical intervention | Appendix perforation, diffused peritonitis or peri appendiceal abscess, intra-abdominal free air, and other causes of acute abdomen, such as urological and gynaecological diseases, contraindication for colonoscopy, and allergy to contrast media |
| M. Kim | | Age 5-18, abdominal pain ≤5 days, pain score of 5 or higher on a vertical visual  analogue scale (VAS), need for surgical consultation | Hypotension, allergy to morphine, suspected pregnancy, prior abdominal surgery, refusal of analgesia, history of sickle cell disease, inflammatory bowel disease, suspected biliary or pancreatic problems |
| H. Kokki | | 4-15 years, undifferentiated acute abdominal pain of less than 7 days' duration, a pain score of 5 cm or higher on a 10-cm-long visual analogue scale | Abdominal trauma,  asthma, hypotension,  known contraindication to oxycodone, and analgesic use prior to ED arrival |
| N. Poonai | | Age 8–17, self-described colicky abdominal pain (≥40 mm VAS at least intense point) | Unable to swallow pills/communicate, received meds <6h prior, suspected surgical/medical pathology, hypersensitivity, nonverbal, trauma, pregnancy, etc. |
| K. Quinn | | Age 3-17 years, weight less  than 64 kg, acute moderate to severe  pain, NRS ≥6 or equivalent Wong-Baker FACES Pain Scale, sufficient intensity to require opioid analgesia as determined by the treating ED attending physician. | Facial trauma or any abnormality  of the nasal anatomy, circulatory insufficiency, developmental delay, head trauma/increased intracranial pressure/altered consciousness, known allergy to fentanyl or ketamine, inability to provide pain scale assessment, and receipt of opioid pain medication immediately before arrival to the ED. |

**Supplementary Table S2: classification of included trials according to the population enrolled**

| **Author** | **Population enrolled** | **Basis for classification (inclusion criteria)** |
| --- | --- | --- |
| **Adult studies** |  |  |
| D. Aghamohammadi | Undifferentiated | Acute surgical abdomen awaiting urgent surgery |
| O. Agodirin | Undifferentiated | Acute abdomen |
| J. Antevil | Suspected biliary disease | Acute right upper quadrant or persistent epigastric pain |
| J. Bartfield | Undifferentiated | Abdominal pain requiring intravenous fentanyl |
| A. Birnbaum | Undifferentiated | Abdominal pain requiring intravenous opioid analgesia |
| A. Chang | Undifferentiated | Acute pain warranting intravenous opioids |
| E. Chinn | Undifferentiated | Acute severe abdominal pain warranting opioid treatment |
| T. Deaton | Undifferentiated | Acute non-injury abdominal pain |
| J. Gallagher | Undifferentiated | Atraumatic abdominal pain warranting opioid analgesia |
| A. Giacosa | Acute diarrhoea | Acute diarrhoea of up to five days |
| K. Kanesan | Undifferentiated | Severe non-traumatic acute abdominal pain |
| K. Kang | Appendicitis | Suspected acute appendicitis with right lower quadrant pain |
| W. Kruis | Diverticulitis | Diverticulitis with acute pain without complications |
| S. Lovett | Undifferentiated | Acute flank, abdominal, back, musculoskeletal or headache pain |
| M. Mahadevan | Appendicitis | Right lower quadrant pain suggestive of acute appendicitis |
| C. Maity | Acute diarrhoea | Acute diarrhoea within 48 hours with abdominal discomfort |
| B. McMahon | Undifferentiated | Moderate to severe abdominal pain |
| J. Miller | Undifferentiated | Abdominal, flank, low back or extremity pain warranting opioids |
| S. Motov (1) | Undifferentiated | Acute abdominal, flank, back or musculoskeletal pain |
| S. Motov (2) | Undifferentiated | Acute abdominal, flank, back, chest or musculoskeletal pain |
| S. Motov (3) | Undifferentiated | Acute pain considered appropriate for ketorolac |
| S. Motov (4) | Undifferentiated | Acute abdominal, flank, back or musculoskeletal pain, aged 65 and above |
| S. Motov (5) | Undifferentiated | Acute pain requiring oral ibuprofen |
| M. Moussa | Renal colic | Acute renal colic diagnosed by CT |
| J. Mozafari | Renal colic | Renal colic confirmed by ultrasound or CT |
| S. Narang | Acute diarrhoea | Acute infectious diarrhoea with moderate to severe abdominal pain |
| T. Nguyen | Undifferentiated | Acute painful conditions considered appropriate for ketamine |
| H. Oguzturk | Undifferentiated | Non-traumatic abdominal pain of less than 72 hours |
| V. Ojetti | Diverticulitis | CT-confirmed acute uncomplicated diverticulitis |
| C. Petruzziello | Diverticulitis | CT-confirmed acute uncomplicated diverticulitis |
| J. Remington-Hobbs | Undifferentiated | Abdominal pain in adults |
| B. Sin | Undifferentiated | Acute pain of up to 15 days |
| S. Thomas | Undifferentiated | Undifferentiated abdominal pain of less than 72 hours |
| C. Yildirim | Acute diarrhoea | Watery stools with abdominal pain |
| C. Zhu | Undifferentiated | Acute gastric or intestinal spasm-like pain without organic cause |
| **Pediatric studies** |  |  |
| B. Bailey | Appendicitis | Right lower quadrant pain with presumptive appendicitis |
| G. Cozzi | Undifferentiated | Moderate to severe acute abdominal pain |
| R. Green | Undifferentiated | Non-traumatic abdominal pain warranting surgical consultation |
| J. Kang | Appendicitis | Acute uncomplicated appendicitis |
| M. Kim | Undifferentiated | Abdominal pain requiring surgical consultation |
| H. Kokki | Undifferentiated | Undifferentiated acute abdominal pain |
| N. Poonai | Undifferentiated | Colicky abdominal pain |
| K. Quinn | Undifferentiated | Acute moderate to severe pain requiring opioid analgesia |

*Trials are classified by the diagnostic status of the population at enrolment. Undifferentiated denotes recruitment on the basis of the symptom and its severity, without an established diagnosis.*

*Ten trials recruited patients with acute pain at mixed anatomical sites, with abdominal pain as one of several eligible locations: S. Lovett, J. Miller, S. Motov (1) to (5), T. Nguyen, B. Sin and K. Quinn.*

**Search string:**

Pubmed:

("Abdominal Pain"[MeSH Terms] OR "acute abdominal pain"[Title/Abstract] OR "visceral pain"[Title/Abstract] OR "acute visceral pain"[Title/Abstract])

AND ("diagnosis"[Title/Abstract] OR "assessment"[Title/Abstract] OR "evaluation"[Title/Abstract] OR "measurement"[Title/Abstract] OR "pain score"[Title/Abstract] OR "pain tool"[Title/Abstract])

AND ("acute disease"[MeSH Terms] OR "acute"[Title/Abstract])

NOT ("Pancreatitis, Acute"[MeSH Terms] OR "acute pancreatitis"[Title/Abstract])

Included clinical studies and randomized trials since year 2000

N = 70 (22/01 2025)

Embase:

('acute abdominal pain':ti OR 'acute visceral pain':ti OR 'abdominal pain'/exp)

AND ('acute pain'/exp OR 'acute':ti AND 'pain':ti)

AND ('randomized controlled trial'/de OR 'controlled clinical trial'/de)

AND ('pain measurement'/exp OR 'pain score':ti,ab OR 'diagnosis'/exp OR 'visual analog scale':ti,ab OR 'numeric rating scale':ti,ab)

AND ([2000-2025]/py)

AND [english]/lim

NOT ('chronic pain'/exp OR 'chronic abdominal pain':ti,ab OR 'somatic pain'/exp OR 'postoperative pain'/exp OR 'surgical procedure'/exp OR 'acute pancreatitis'/exp OR 'acute pancreatitis':ti,ab OR 'irritable bowel syndrome'/exp OR 'IBS':ti,ab)

N = 243 (03/02 2025)

Cochrane Library: acute abdominal pain

N = 19 (03/02/2025)

Scopus:

(TITLE("acute abdominal pain") OR TITLE("acute visceral pain") OR TITLE-ABS-KEY("abdominal pain"))

AND

(TITLE-ABS-KEY("acute pain") OR (TITLE("acute") AND TITLE("pain")))

AND

(TITLE-ABS-KEY("randomized controlled trial") OR TITLE-ABS-KEY("controlled clinical trial"))

AND

(TITLE-ABS-KEY("pain measurement") OR TITLE-ABS-KEY("pain score") OR TITLE-ABS-KEY("diagnosis")

OR TITLE-ABS-KEY("visual analog scale") OR TITLE-ABS-KEY("numeric rating scale"))

AND

(PUBYEAR > 1999 AND PUBYEAR < 2026)

AND

(LIMIT-TO(LANGUAGE, "English"))

AND

NOT (TITLE-ABS-KEY("chronic pain") OR TITLE-ABS-KEY("chronic abdominal pain") OR TITLE-ABS-KEY("somatic pain")

OR TITLE-ABS-KEY("postoperative pain") OR TITLE-ABS-KEY("surgical procedure")

OR TITLE-ABS-KEY("acute pancreatitis") OR TITLE-ABS-KEY("irritable bowel syndrome") OR TITLE-ABS-KEY("IBS"))

N = 95 (03/02 2025)

Web of Science

(((((((TS=(abdominal pain)) OR TS=(acute abdominal pain)) OR TS=(visceral pain)) OR TS=(acute visceral pain)) AND ((((((TS=(assessment)) OR TS=(evaluation)) OR TS=(diagnosis)) OR TS=(measurement)) OR TS=(pain score)) OR TS=(pain tool))) AND ((TS=(acute disease)) OR TS=(acute))) NOT ((((TS=(acute pancreatitis)) OR TS=(postoperative pain)) OR TS=(post-operative pain)) OR TS=(post operative pain))) AND TS=(randomized controlled trial)

N=130 (04-02-2025)
